# Association of low muscle mass and obesity with increased all‐cause and cardiovascular disease mortality in US adults

**DOI:** 10.1002/jcsm.13397

**Published:** 2023-12-18

**Authors:** Donghyun Kim, Junghoon Lee, Raekil Park, Chang‐Myung Oh, Shinje Moon

**Affiliations:** ^1^ Department of Cardiology Chonbuk National University Hospital Jeonju Korea; ^2^ Department of Internal Medicine, Hallym University Kangnam Sacred Heart Hospital Hallym University College of Medicine Seoul Korea; ^3^ Department of Biomedical Science and Engineering Gwangju Institute of Science and Technology Gwangju Korea

**Keywords:** Cardiovascular disease, Central obesity, Metabolic syndrome, Sarcopenia

## Abstract

**Background:**

Sarcopenic obesity, defined as the coexistence of low muscle mass and high adiposity, is associated with cardiovascular disease (CVD) and mortality. However, to what extent sarcopenia contributes to these risks independently or in conjunction with other cardiovascular risk factors remains unclear. This study aimed to investigate the association of low muscle mass, central obesity (COB), metabolic abnormalities, and their combinations with CVD and mortality risk.

**Methods:**

This cross‐sectional analysis used data from the National Health and Nutrition Examination Survey 1999–2006 and 2011–2018. Participants aged >20 years and with reported whole‐body dual X‐ray absorptiometry data were included. Participants were divided into eight groups based on low muscle mass, metabolic abnormalities, and COB status.

**Results:**

The mean age of participants was 55 years, and 50.4% of participants were male. Low muscle mass was observed in 2472 (14.6%) out of 16 839 participants. Among the eight groups, the metabolically unhealthy COB group with low muscle mass had the highest hazard ratio (HR) for all‐cause mortality (HR, 2.00; 95% CI, 1.56–2.56; *P* < 0.001), whereas the metabolically healthy COB group with low muscle mass had the highest HR for CVD mortality (HR, 3.18; 95% CI, 1.53–6.65; *P* = 0.001). The mediation analysis showed that low muscle mass directly increased the risk of both all‐cause mortality (HR, 1.56; 95% CI, 1.35–1.79; *P* < 0.001) and CVD mortality (HR, 1.80; 95% CI, 1.40–2.31; *P* < 0.001). Additionally, subgroup analysis revealed that low muscle mass significantly increased the risk of all‐cause and CVD mortality in participants without a prior CVD history and those with diabetes mellitus.

**Conclusions:**

Low muscle mass is an independent risk factor for all‐cause and CVD mortality, especially in individuals with metabolic abnormalities and COB.

## Introduction

Sarcopenia, a condition characterized by the loss of muscle mass and strength, has been a growing concern among clinical researchers due to its association with various long‐term conditions.^1^ Interestingly, recent research suggests that sarcopenia may play a role in the ‘obesity paradox’, a phenomenon in which overweight individuals may exhibit lower mortality rates than those with normal body mass index (BMI). The underlying mechanism behind this relationship is not yet fully understood; however, it has been suggested that the loss of muscle mass and function associated with sarcopenia may contribute to the increased mortality risk seen in individuals with a normal BMI.^2^

It is commonly assumed that obesity provides protection against sarcopenia, as individuals with higher body fat seem to retain more muscle mass. However, studies have shown that obesity can impair muscle function, leading to functional limitations.^3^, ^4^ Furthermore, it is important to note that sarcopenia can occur in individuals with obesity, a condition referred to as sarcopenic obesity. These individuals may have a high BMI but poor lean body mass, leading to increased disability, immobility and metabolic dysfunction.^5^, ^6^ The prevalence of sarcopenic obesity in older adults has become a significant concern, given its negative impact on quality of life, physical function and mortality.^7^, ^8^ Therefore, clinicians and researchers must recognize and address sarcopenic obesity as a distinct clinical entity to improve the health outcomes and enhance the quality of life of older adults.

Frailty is another similar, albeit different, term referring to a clinical syndrome associated with aging. While there exists no consensus on the definition of frailty, it is theoretically defined as a clinically recognizable state of increased vulnerability resulting from age‐related decline in multiple physiological systems.^9^ On the one hand, the frailty phenotype specifies frailty as poor performance in three out of the following five criteria: weight loss, exhaustion, weakness, slowness and a lack of activity. On the other hand, the frailty index is a ratio of the number of deficits accumulated by an individual divided by all deficits measured.^10^, ^11^ Sarcopenia and frailty overlap, particularly with respect to the physical aspects of the frailty phenotype, such as low grip strength, gait speed, and muscle mass.^12^

Given that traditional cardiovascular risk factors are often present in individuals with sarcopenic obesity, the extent to which sarcopenia alone or in combination with other risk factors contributes to cardiovascular disease (CVD) and mortality risk remains unclear. To address this knowledge gap, we conducted a study using data from the National Health and Nutrition Examination Survey (NHANES) to investigate the association of low muscle mass, central obesity (COB), metabolic abnormalities, and their combinations with CVD and mortality risk. Specifically, we sought to determine whether low muscle mass alone or in conjunction with COB and metabolic abnormalities is independently associated with an increased risk of CVD and mortality. Additionally, we evaluated the association between sarcopenia and frailty in relation to CVD and mortality and also performed subgroup analysis to investigate whether the association of low muscle mass with increased CVD and mortality risk differs according to the presence or absence of diabetes mellitus (DM) or prior CVD history. Such subgroup analysis enables us to explore potential effect modifiers and to provide a more nuanced understanding of the relationship between low muscle mass and CVD/mortality risk in specific patient populations. Our findings could have important implications for the management and prevention of CVD and mortality in individuals with sarcopenic obesity.

## Methods

### Study population

The NHANES is a cross‐sectional survey including questions related to health and nutrition, medical, dental, physical measurements, and laboratory analysis with a representative sample of the US population.^13^ We acquired baseline data from four NHANES cycles 1999–2006 and 2011–2018. These baseline data were connected to the mortality data from the National Death Index for the longitudinal study.^14^

### Measurement

The uppermost lateral border of the ilium was measured using a flexible measuring tap to determine waist circumference (WC). The participant's blood pressure (BP) was measured three times after a minimum of 5 min of rest while sitting, and the mean of the three readings was taken. Enzymatic methods were used to measure fasting blood glucose and cholesterol levels. The NHANES Laboratory Procedures Manual has more information on sample collection and testing methodologies.^15^ Whole‐body dual X‐ray absorptiometry was performed using a Hologic QDR 4500A fan‐beam X‐ray bone densitometer (Hologic Inc., Marlborough, MA, USA) in NHANES 1999–2006 and Hologic Discovery model A densitometers in NHANES 2011–2018. Total and regional body compositions were also assessed using dual X‐ray absorptiometry.

### Definition of COB, metabolic disorder, and low muscle mass

COB was defined as a WC > 102 cm in men and >88 cm in women based on the revised National Cholesterol Education Program‐Adult Treatment Panel III criteria (NCEP‐ATP III criteria) for metabolic syndrome.^16^ Because of the lack of a clear definition for metabolically healthy status, we assessed metabolically healthy status using the updated NCEP‐ATP III criteria for metabolic syndrome, which were the most frequently used criteria in prior investigations.^17^, ^18^, ^19^ Metabolic abnormality was defined as having two or more metabolic risk factors, including impaired fasting glucose (i.e., a fasting glucose level >100 mg/dL or a diagnosis of DM), high BP (systolic BP > 130 mmHg and/or diastolic BP > 85 mmHg or a diagnosis of HTN), triglyceride level ≥150 mg/dL, and a high‐density lipoprotein cholesterol level <40 mg/dL in men and <50 mg/dL in women. Appendicular skeletal mass was defined as the sum of the total lean mass, excluding the bone mineral content of both arms and legs, and the appendicular skeletal mass index (ASMI) was defined as the value obtained by dividing the appendicular skeletal mass by the square of the height (m).^20^ Low muscle mass was defined as an ASMI <7 kg/m^2^ in men or <5.5 kg/m^2^ in women according to the European Working Group on Sarcopenia in Older People 2 (EWGSOP2).^21^

### Group definition

All participants were categorized into one of eight groups based on baseline skeletal mass, metabolic health, and obesity status: (1) metabolically healthy (MH)‐normal WC (NW) group with normal muscle mass; (2) MH‐COB group with normal muscle mass; (3) metabolically unhealthy (MU)‐NW group with normal muscle mass; (4) MU‐COB group with normal muscle mass, MH‐normal WC group with low muscle mass; (5) MH‐NW with low muscle mass; (6) MH‐COB group with low muscle mass; (7) MU‐NW group with low muscle mass; and (8) MU‐COB with low muscle mass.

### Study outcomes

Data on all‐cause and CVD mortality and follow‐up duration by months were gathered from public‐use‐linked mortality data at the National Center for Health Statistics, which were based on the probabilistic match between NHANES and National Death Index death certificate records up to December 31, 2019.^14^

### Ethics statement

The study protocol was approved by the institutional review board of Kangnam Sacred Heart Hospital (IRB No. HKS 2020‐01‐020). All NHANES procedures in the United States were authorized by the National Center for Health Statistics' Research Ethics Review Board (NCHS IRB/ERB Protocol Numbers: 1999–2004, Protocol #98‐12, and 2005‐206, Protocol #2005‐06). All participants signed a written informed consent form.

### Definition of covariates

Covariates included age, sex, race/ethnicity, smoking status, alcohol consumption, history of cancer, estimated glomerular filtration rate (eGFR), and metabolic disorders, such as dyslipidaemia, HTN, DM and CVD at the baseline. The questionnaire responses provided information on age, sex, race/ethnicity, smoking status, alcohol intake, and cancer history. The eGFR was calculated using the Modification of Diet in Renal Disease formula. HTN was defined as follows: systolic BP > 140 mmHg, mean diastolic BP > 90 mmHg, or treatment for HTN. A diagnosis of DM was made using fasting blood glucose levels and glycated haemoglobin (HbA1c) levels as follows: fasting blood glucose levels >126 mg/dL, random blood glucose levels >200 mg/dL, and HbA1c levels >6.5%, or treatment for DM. Dyslipidaemia was defined as fasting total cholesterol ≥240 mg/dL or treatment for dyslipidaemia. To investigate the history of CVD episodes, a standardized questionnaire was employed. Participants with angina pectoris, coronary heart disease, myocardial infarction, congestive heart failure, or cerebrovascular disease were regarded as having a history of CVD events. Alcohol intake was measured by asking each participant to quantify the number of drinks consumed, and drinkers were considered if they consumed ≥12 drinks in their lifetime and currently drinking (≥1 drink per month).

The frailty index was constructed by selecting 46 variables commonly included in 10 NHANES cycles according to standard procedures^22^, ^23^ and was determined by dividing the number of present health deficits by the number of measured health deficits.^23^ Table S1 presents the frailty index in detail. Individuals with available data on ≥40 out of the 46 frailty index items were included in the analysis.

### Statistical analysis

Continuous and categorical variables of basic demographic characteristics, underlying diseases, anthropometric indices, and blood test results are displayed using the mean with the standard deviation and frequency (%), respectively. The hazard ratios (HRs) for all‐cause and CVD mortality were determined using multiple Cox regression analysis adjusted for the following confounding variables: age, sex, race, smoking status, alcohol consumption, eGFR, COB, history of cancer, HTN, DM, dyslipidaemia and previous CVD events. Follow‐up duration was computed as the time from the first anthropometric and clinical measurement to death or the last follow‐up (31 December 2019).^24^ The graphical association between HR for each obesity parameter and mortality was assessed using restricted cubic spline (RCS) plots with four knots.

To investigate the direct effect of low muscle mass on mortality independent of the effects of metabolic health status, we conducted a regression‐based causal mediation analysis using the package ‘Regmedint’ developed by Yoshida et al.^25^ It is the R counterpart to mediation macro in SAS by Valeri and VanderWeele.^26^, ^27^ We calculated the total natural indirect effect (TNIE), pure natural indirect effect (PNIE), total natural direct effect (TNDE), pure natural direct effect (PNDE) and total effect (TE) on mortality. Additionally, we adopted the 2013 ACC/AHA guideline on the assessment of cardiovascular risk in determining the atherosclerotic CVD (ASCVD) risk score.^28^ Statistical analysis was performed using International Business Machines (Armonk, NY, USA) (IBM) Statistical Package for Social Sciences version 24.0 (IBM Corporation, Armonk, NY, USA) and R version 3.1.0 (R Foundation for Statistical Computing, Vienna, Austria; www.r‐project.org), with statistical significance set a *P*‐value of <0.05.

## Results

### Baseline characteristics of the participants

The present study enrolled a total of 16 839 participants from the NHANES 1999–2006 and 2011–2018 database. The mean age of the participants was 45.3 years, with men comprising 50.1% of the total population (Figure S1). The demographic and clinical characteristics of the participants with and without low muscle mass are summarized in Table 1. The prevalence of low muscle mass was found to be significantly higher in women (20.7%) compared to men (8.7%). Participants with low muscle mass demonstrated lower BMI and WC measurements compared to those without low muscle mass. In addition, participants with low muscle mass exhibited lower levels of fasting glucose, HbA1c and triglycerides, with higher values of high‐density lipoprotein cholesterol, in comparison with those with normal muscle mass. Interestingly, the prevalence of metabolic disorders such as DM, metabolic syndrome, COB and metabolically unhealthy status was significantly lower in participants with low muscle mass than in those without. However, individuals with low muscle mass showed a higher frailty index score, higher all‐cause and cardiovascular mortality rates, and higher prevalence of dyslipidaemia, hypertension and previous CVD events than their non‐sarcopenic counterparts.

**Table 1:** Overall characteristics of the participants according to muscle mass

Variables	Normal	Low muscle mass	*P*‐value
	(*N* = 14 367)	(*N* = 2472)	
Age, years	44.2 ± 14.6	51.7 ± 18.7	<0.001
Female sex, *n* (%)	6655 (46.3%)	1740 (70.4%)	<0.001
Race/ethnicity, *n* (%)			<0.001
Hispanic	3656 (25.4%)	513 (20.8%)	
Non‐Hispanic White	6106 (42.5%)	1421 (57.5%)	
Non‐Hispanic Black	3020 (21.0%)	156 (6.3%)	
Other races	1585 (11.0%)	382 (15.5%)	
Smokers, *n* (%)	6269 (43.6%)	1108 (44.8%)	0.281
Drinkers, *n* (%)	10 070 (70.1%)	1560 (63.1%)	<0.001
BMI, kg/m^2^	29.4 ± 5.8	22.4 ± 3.0	<0.001
Waist circumference, cm	99.2 ± 14.3	83.8 ± 10.4	<0.001
Systolic BP, mmHg	122.4 ± 17.3	123.9 ± 22.1	0.002
Diastolic BP, mmHg	72.4 ± 11.9	69.8 ± 12.3	<0.001
Glucose, mg/dL	107.6 ± 36.0	102.8 ± 35.1	<0.001
HbA1C, %	5.7 ± 1.1	5.5 ± 1.0	<0.001
Total cholesterol, mg/dL	198.5 ± 43.9	201.8 ± 42.1	<0.001
Triglycerides, mg/dL	154.2 ± 156.2	124.4 ± 92.8	<0.001
HDL, mg/dL	51.1 ± 14.9	60.6 ± 17.1	<0.001
LDL, mg/dL	113.8 ± 38.9	114.3 ± 36.0	0.641
GFR, mL/min/1.73 m^2^ (MDRD)	114.5 ± 22.4	114.9 ± 29.2	0.564
ASMI	8.2 ± 1.5	5.7 ± 0.7	<0.001
Previous CVD events, *n* (%)	992 (6.9%)	265 (10.7%)	<0.001
Diabetes mellitus, *n* (%)	1885 (13.1%)	234 (9.5%)	<0.001
Medication for DM	1110 (7.7%)	153 (6.1%)	0.006
Hypertension, *n* (%)	5573 (38.8%)	1046 (42.3%)	0.001
Medication for hypertension	3202 (22.3%)	674 (27.3%)	<0.001
Dyslipidaemia, *n* (%)	5970 (41.6%)	1088 (44.0%)	0.023
Medication for dyslipidaemia	1596 (11.1%)	329 (13.3%)	0.002
Metabolic syndrome*, *n* (%)	5014 (34.9%)	512 (20.7%)	<0.001
Central obesity, *n* (%)	8093 (56.3%)	551 (22.3%)	<0.001
Metabolic abnormality^†^, *n* (%)	6143 (65.3%)	803 (50.7%)	<0.001
Frailty index	0.106 ± 0.090	0.128 ± 0.106	<0.001
Malignancies at baseline survey, *n* (%)	771 (5.4%)	263 (10.6%)	<0.001
All‐cause mortality, *n* (%)	1438 (10.0%)	671 (27.2%)	<0.001
CVD mortality, *n* (%)	444 (3.1%)	209 (8.5%)	<0.001

Abbreviations: ASMI, appendicular skeletal muscle index; BMI, body mass index; BP, blood pressure; CVD, cardiovascular disease; FMI, fat mass index; GFR, glomerular filtration rate; HDL, high density cholesterol; MDRD, Modification of Diet in Renal Disease study.

* Metabolic syndrome defined by the revised NCEP‐ATPIII criteria.

† Metabolic abnormality was defined as having two or more metabolic syndrome components of the revised NCEP‐ATP III criteria, except for central obesity.

### Effects of low muscle mass on mortality risk according to COB and metabolic abnormalities

**Figure 1:**
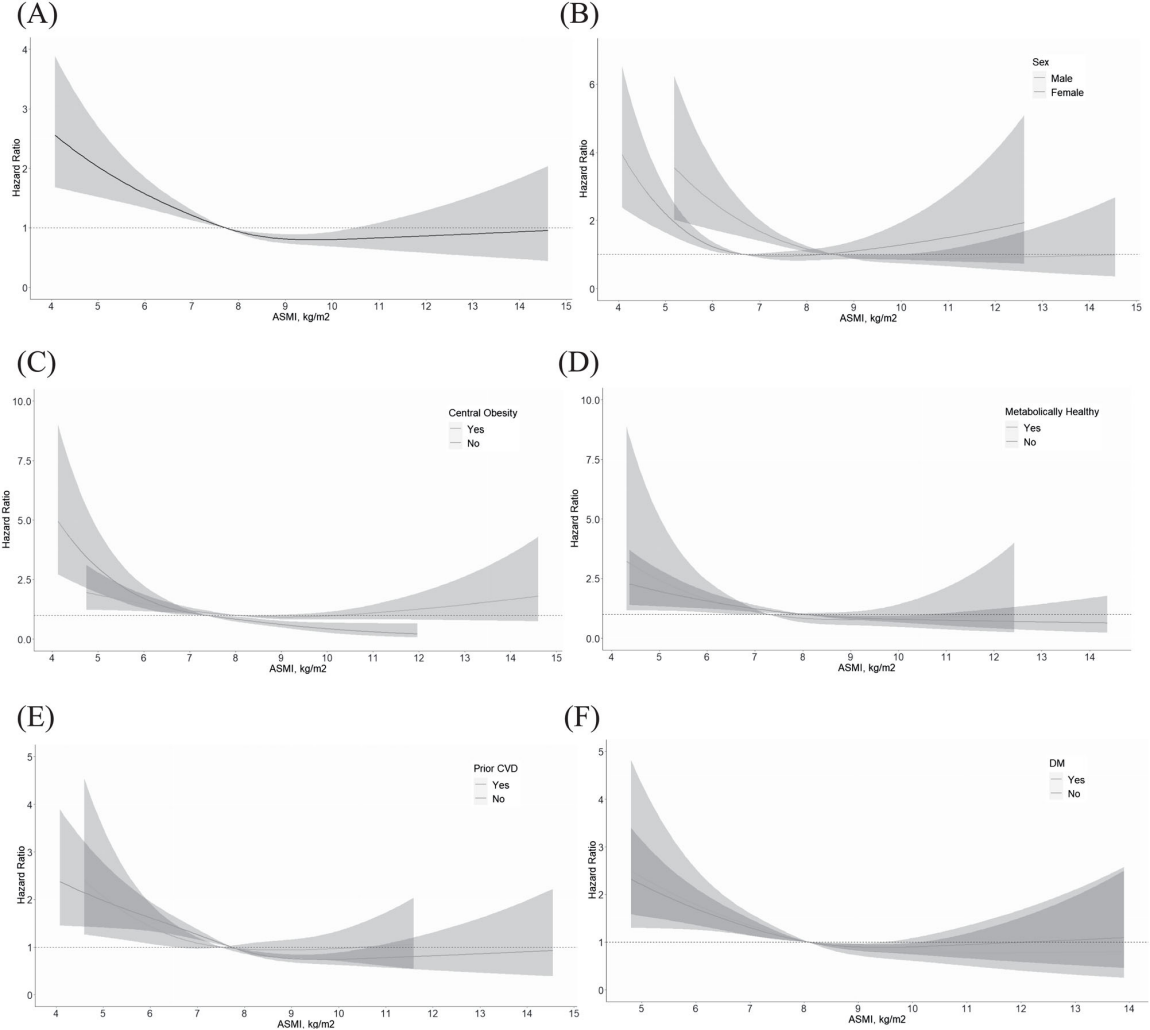
Hazard ratio according to the continuous value of ASMI. (A) ASMI and HR of total participants, (B) ASMI and HR according to sex, (C) ASMI and HR according to presence of COB, (D) ASMI and HR according to presence of metabolic abnormalities, (E) ASMI and HR according to presence of CVD, (F) ASMI and HR according to presence of DM adjusted for age, sex, race, smoking status, alcohol consumption, eGFR, COB, history of cancer, HTN, DM, dyslipidaemia, and past CVD events. Abbreviations: ASMI, appendicular skeletal muscle index; COB, central obesity; CVD, cardiovascular disease; HR, hazard ratio.

**Figure 2:**
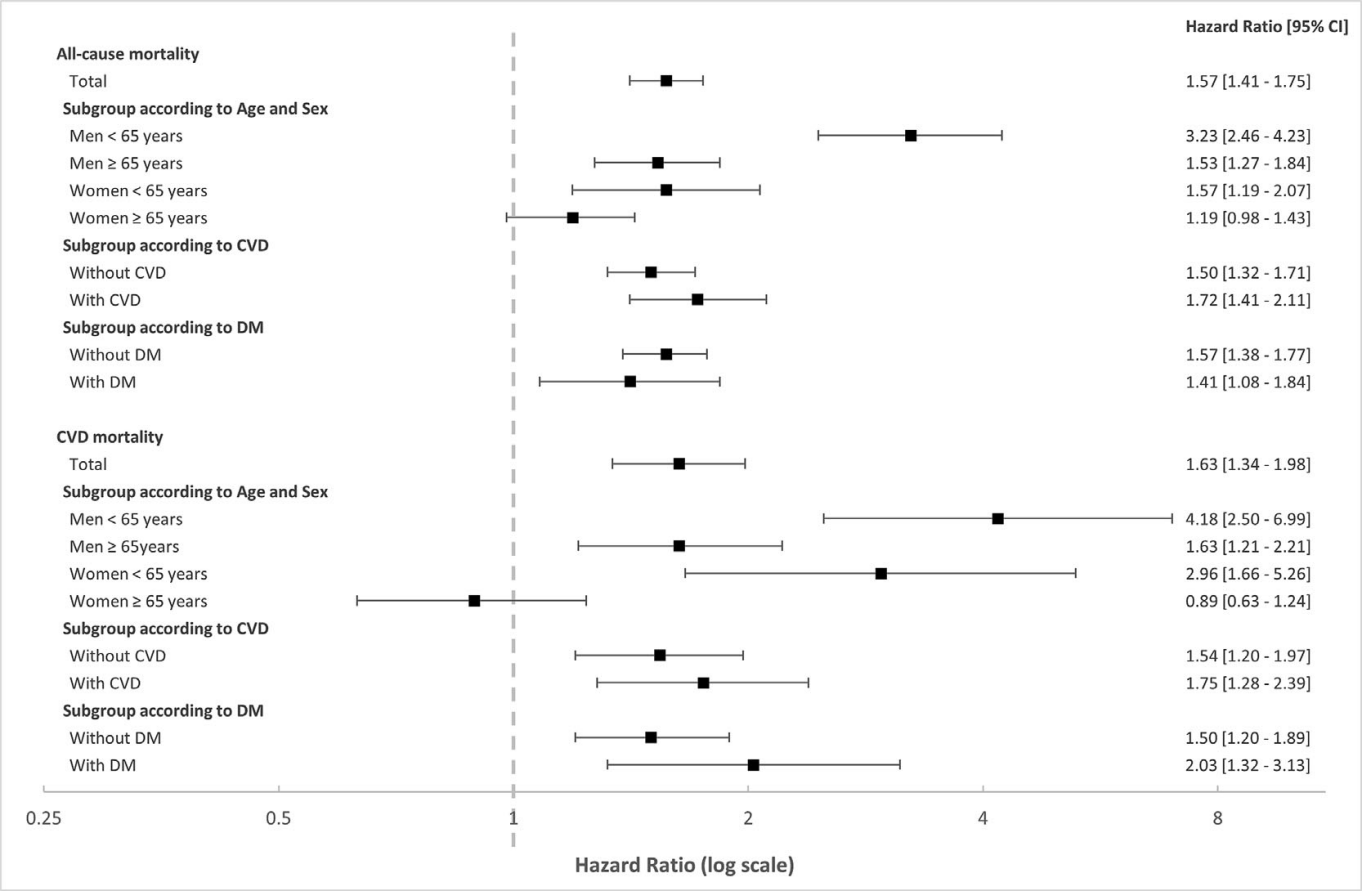
Hazard ratios for all‐cause and CVD mortality according to the low muscle mass. Adjusted for age, sex, race, smoking status, alcohol consumption, eGFR, COB, history of cancer, HTN, DM, dyslipidaemia, and past CVD events.

The association of low muscle mass with all‐cause and CVD mortality was assessed using a multiple Cox regression model. The RCS plot analysis indicated a continuous association between low ASMI and an increased risk of all‐cause mortality in both men and women (Figure 1A,B). The adjusted HR for all‐cause and CVD mortality was 1.57 (95% CI, 1.41–1.75) and 1.63 (95% CI, 1.34–1.98), respectively (Figure 2). Subgroup analysis by sex and age revealed that low muscle mass was significantly associated with a higher risk of all‐cause and CVD mortality in men aged <65 years, men aged >65 years and women aged <65 years (Figure 2 and Figure S3).

**Figure 3:**
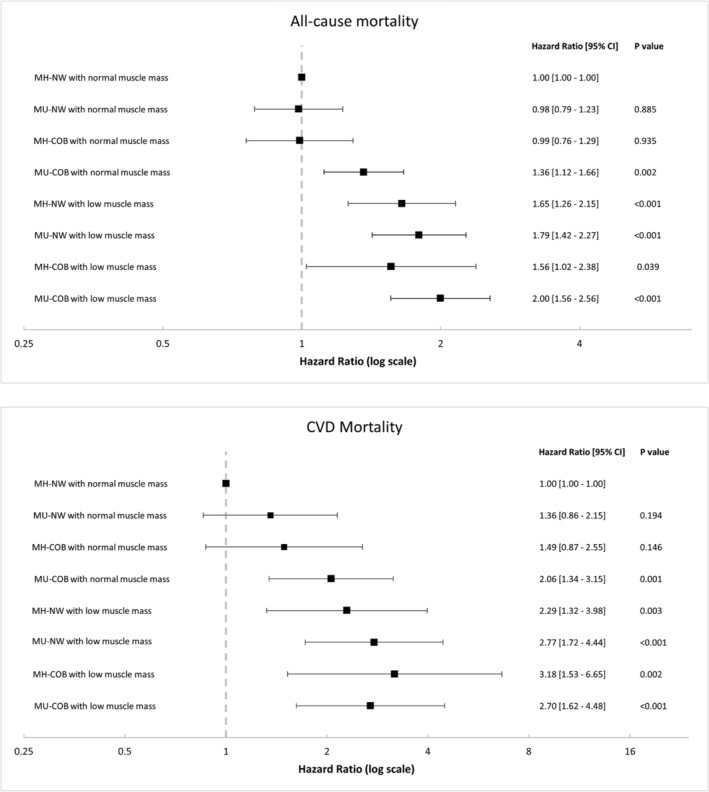
Effects of low muscle mass on mortality risk according to central obesity and metabolic abnormalities. Adjusted for age, sex, race, smoking status, alcohol consumption, eGFR, COB, and history of cancer. Abbreviations: COB, central obesity; CVD, cardiovascular disease; MU, metabolically unhealthy status; MU_COB, metabolically unhealthy status with central obesity.

In order to comprehensively evaluate the impact of low muscle mass on mortality and metabolic status, we categorized study participants into eight groups based on their baseline skeletal mass, metabolic health and obesity status. Kaplan–Meier analysis revealed that individuals with both metabolic abnormality and low muscle mass had a substantially higher risk of all‐cause and CVD mortality than those in other groups (Figure S2). To further explore the relationship between low muscle mass and mortality risk among the eight groups, multiple Cox regression analysis was performed (Figure 3). Our analysis showed that the MU‐COB group with low muscle mass exhibited the highest risk for all‐cause mortality (HR, 2.00; 95% CI, 1.56–2.56), whereas the MH‐COB group with low muscle mass demonstrated the highest HR for CVD mortality (3.18; 95% CI, 1.53–6.65). Notably, the MH‐COB group and the MU‐NW group with normal muscle mass did not exhibit a significant increase in risk for all‐cause and CVD mortality, as compared with the MH‐NW group with normal muscle mass. However, a significant association between low muscle mass and all‐cause mortality risk was observed in the MH‐COB and MU‐NW groups with low muscle mass.

**Figure 4:**
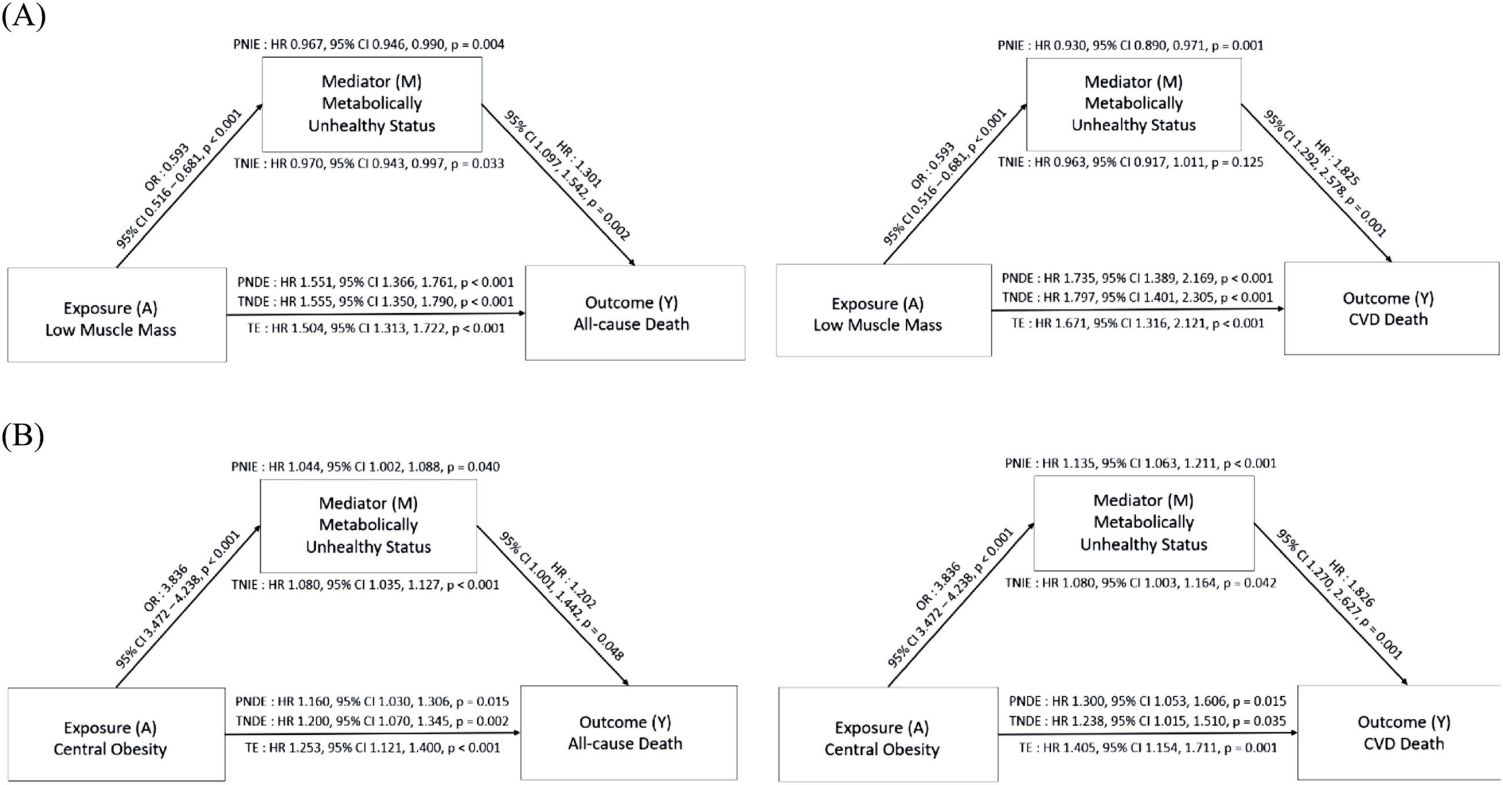
Mediation analysis of effect of low muscle mass and central obesity through metabolically unhealthy status on all‐cause and CVD mortality. (A) Low muscle mass, (B) central obesity. Adjusted for age, sex, race, smoking status, alcohol consumption, eGFR, low muscle mass, and central obesity. Abbreviations: PNDE, pure natural direct effect; PNIE, pure natural indirect effect; TE, total effect; TNDE, total natural direct effect; TNIE, total natural indirect effect.

To further explore the relationships between low muscle mass, COB, metabolically unhealthy status and mortality, a mediation analysis was conducted. The results are presented in Figure 4. The total effect of low muscle mass on all‐cause mortality was significant (HR, 1.50; 95% CI, 1.31–1.72). Low muscle mass was associated with a decreased prevalence of metabolic abnormality (odds ratio [OR], 0.59; 95% CI, 0.52–0.68) and a reduced risk of mortality through metabolically unhealthy status (PNIE: HR, 0.97; 95% CI, 0.95–0.99). However, despite this indirect beneficial effect on mortality, low muscle mass was directly associated with an increased risk of all‐cause mortality (TNDE: HR, 1.56; 95% CI, 1.35–1.79) (Figure 4A). In contrast, COB was found to increase the risk of all‐cause mortality mediated by metabolically unhealthy status (PNIE: HR, 1.04; 95% CI, 1.00–1.09; *P* = 0.04). The mediation analysis of CVD mortality demonstrated comparable findings (Figure 4B).

### Effects of low muscle mass on mortality risk according to frailty status

**Figure 5:**
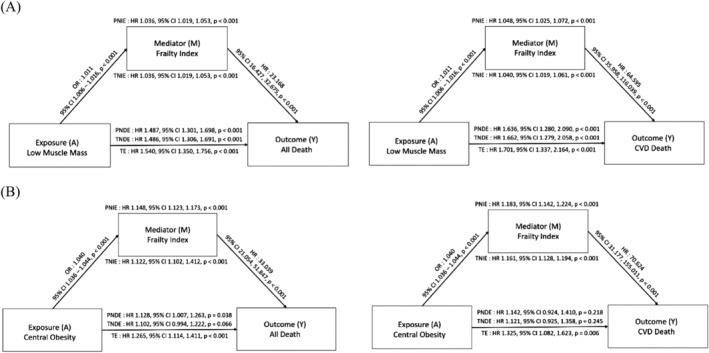
Mediation analysis of effect of low muscle mass and central obesity through frailty status on all‐cause and CVD mortality. (A) Low muscle mass, (B) central obesity. Adjusted for age, sex, race, smoking status, alcohol consumption, eGFR, low muscle mass, and central obesity. Abbreviations: PNDE, pure natural direct effect; PNIE, pure natural indirect effect; TE, total effect; TNDE, total natural direct effect; TNIE, total natural indirect effect.

By conducting a mediation analysis, we presented a conceptual model in which low muscle mass and COB were associated directly and indirectly with mortality through frailty (Figure 5). Low muscle mass had a significant direct effect on all‐cause and CVD mortality (Figure 5A) and was also associated with an increased frailty index score, which was related to all‐cause and CVD mortality. Low muscle mass indirectly increased the risk of all‐cause and CVD mortality via frailty. Nevertheless, frailty was estimated to mediate only 9.8% of all‐cause mortality risk and 9.3% of CVD mortality risk, with low muscle mass predominantly contributing directly to the increased mortality risk. In contrast, COB did not have a direct effect on mortality; instead, it indirectly increased the mortality risk through the mediation of frailty (Figure 5B).

### Effects of low muscle mass on mortality risk according to previous CVD and CVD risk score

In the subgroup analysis of patients without a prior CVD history, our study's results indicate that low muscle mass is significantly associated with an elevated risk of both all‐cause mortality (HR, 1.50; 95% CI, 1.32–1.71) and CVD mortality (HR, 1.54; 95% CI, 1.20–1.97; Figure 2). The results from the RCS plot analysis further support the notion that low ASMI is a strong predictor of all‐cause mortality, irrespective of the patients' CVD status (Figure 1E).

**Figure 6:**
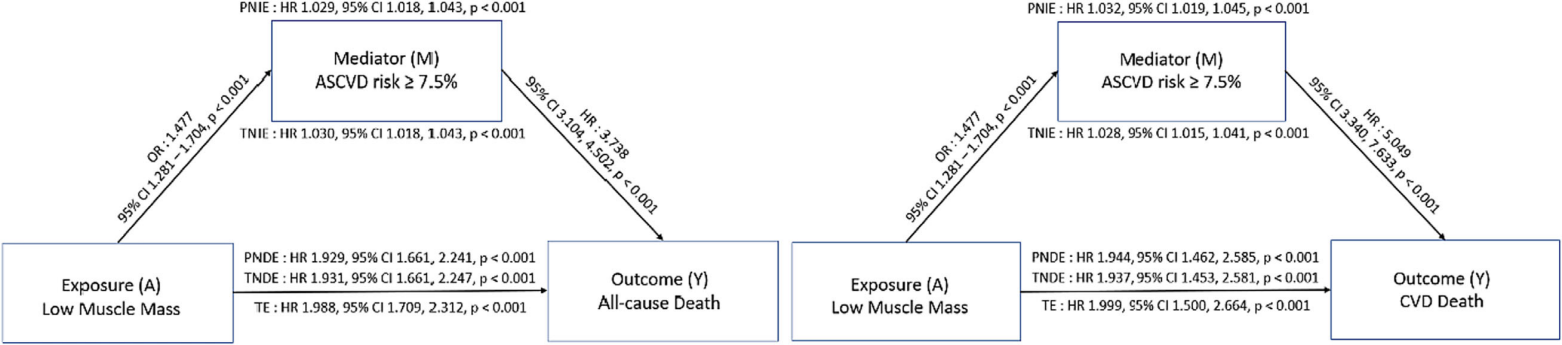
Mediation analysis of effect of low muscle mass through ASCVD risk in individuals without previous CVD on all‐cause and CVD mortality. Adjusted for alcohol consumption, eGFR, central obesity, and history of cancer. Abbreviations: PNDE, pure natural direct effect; PNIE, pure natural indirect effect; TE, total effect; TNDE, total natural direct effect; TNIE, total natural indirect effect.

In the mediation analysis, the total effect of low muscle mass on all‐cause mortality was significant (HR, 1.988; 95% CI, 1.709–2.312). Patients with low muscle mass had a higher ASCVD risk score (≥7.5%), indicating a higher likelihood of ASCVD development (OR, 1.477; 95% CI, 1.281–1.704). Furthermore, low muscle mass increased the risk of all‐cause mortality both directly and indirectly through a high ASCVD risk score (PNIE: HR, 1.03; 95% CI, 1.02–1.04; TNDE: HR, 1.93; 95% CI, 1.66–2.25). These findings suggest that the detrimental effect of low muscle mass on mortality risk may be partly mediated by an increased risk of ASCVD. The mediation analysis of CVD mortality showed similar results, with low muscle mass being associated with an increased risk of CVD mortality both directly and indirectly through a high ASCVD risk score (Figure 6).

### Effects of low muscle mass on mortality risk according to DM

Next, we performed a multiple Cox regression analysis that took into account several potential confounding factors, such as age, sex, race, smoking status, alcohol consumption, COB, history of cancer, HTN, dyslipidaemia, previous CVD events, duration of DM, microvascular complications (nephropathy or retinopathy), and haemoglobin A1c (HbA1c). In people with DM, low muscle mass was found to be associated with a higher risk of all‐cause mortality (HR, 1.41; 95% CI, 1.08–1.84) and CVD mortality (HR, 2.03; 95% CI, 1.32–3.13) (Figure 2). Furthermore, the RCS plot analysis demonstrated that low ASMI was consistently linked to an increased risk of all‐cause mortality (Figure 1F).

**Figure 7:**
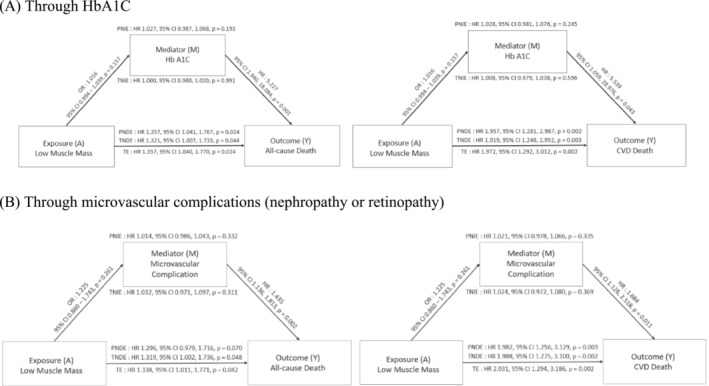
Mediation analysis of effect of low muscle mass through HbA1c and microvascular complications in patients with DM on all‐cause and CVD mortality. Adjusted for age, sex, race, smoking status, alcohol consumption, central obesity, history of cancer, HTN, dyslipidaemia, previous CVD events, duration of DM, microvascular complications and haemoglobin A1c (HbA1c). Abbreviations: PNDE, pure natural direct effect; PNIE, pure natural indirect effect; TE, total effect; TNDE, total natural direct effect; TNIE, total natural indirect effect.

In the subgroup analysis of people with DM, the mediation analysis revealed that low muscle mass had a direct impact on the risk of all‐cause and CVD mortality rather than indirectly mediated by HbA1c or microvascular complications (Figure 7). Notably, low muscle mass did not exhibit any significant association with high HbA1c or microvascular complications. Therefore, our results suggest that low muscle mass may play a direct role in the pathogenesis of all‐cause and CVD mortality in patients with DM, independent of other confounding factors.

## Discussion

In this study, we investigated the relationship between low muscle mass and mortality risk in individuals with and without metabolic abnormalities and COB. To the best of our knowledge, this nationwide population‐based study was the first to compare mortality risk in participants grouped by baseline skeletal mass, metabolic health, and obesity status and identified the direct effect of low muscle mass on mortality by mediation analysis, despite a low prevalence of COB and metabolic abnormality in the general population with low muscle mass.

In line with findings from previous studies, we discovered that low muscle mass is significantly associated with mortality. Previous studies have examined the impact of sarcopenia on the risk of functional decline, reduced mobility, frailty, falls, fractures, and hospitalization, all of which may contribute to a higher risk of mortality.^29^, ^30^, ^31^ Our study's subgroup analysis further highlights the importance of evaluating muscle mass, particularly in men and individuals aged <65 years (CVD mortality: HR, 5.07; 95% CI, 2.71–9.48). These differences in survival outcomes between men and women may be attributable to the natural differences in skeletal muscle mass between the sexes.^32^, ^33^

Our findings are consistent with the research conducted by Kristina et al., which highlights that men may experience greater skeletal muscle mass loss and functional attenuation than women. Furthermore, men with similar levels of muscle loss are more likely to be at risk than women.^34^, ^35^ These sex‐based differences may be attributed to the hormonal status of males (testosterone, luteinizing hormone).^36^, ^37^ While sarcopenia is widely recognized as an age‐associated loss of skeletal mass and function, most studies have primarily focused on understanding sarcopenia among older adults.^38^, ^39^ However, it is important to acknowledge that consistent muscular intervention should be pursued from a younger age, as our research suggests that muscle loss may pose a greater risk of mortality in individuals aged <60 years, as opposed to older adults.

The relationship between sarcopenia and negative health outcomes in patients with metabolic abnormalities has garnered significant interest in the scientific community.^40^, ^41^ In this study, we examined the impact of low muscle mass on mortality by categorizing participants based on their baseline skeletal mass, metabolic health, and obesity status. Our results demonstrate that the MH‐COB group with low muscle mass had significantly higher all‐cause and CVD mortality than the MH‐COB group without low muscle mass, suggesting that sarcopenia may serve as a vital prognostic indicator for survival outcomes. Conversely, the MU‐normal WC and MH‐COB groups without low muscle mass did not show any significant risks for all‐cause and CVD mortality. However, the MH‐normal WC group with low muscle mass exhibited a heightened risk for both all‐cause and CVD mortality, indicating that effective interventions are necessary for individuals with low muscle mass, irrespective of their metabolic syndrome components.

Moreover, our findings revealed that participants with metabolic abnormalities and low muscle mass exhibited a greater risk of mortality than the other groups, emphasizing the potential impact of low muscle mass on overall survival in metabolically unhealthy populations. Our study results are consistent with those of Dana et al., who reported that low muscle mass in obese individuals was associated with worse metabolic disorders and an increased risk of mortality.^42^ Taken together, these findings suggest that improving the muscle mass and function may be an important strategy for improving overall health outcomes, particularly in populations with metabolic abnormalities. Our subgroup analysis revealed that low muscle mass increased the risk of both all‐cause and CVD mortality in individuals without a prior CVD history. Furthermore, even in individuals with DM, low muscle mass showed a deleterious impact on both all‐cause and CVD mortality. Based on these findings, we propose that sarcopenia should be considered an independent determinant of mortality risk in clinical evaluations, regardless of conventional risk factors such as metabolic disorders and obesity. Given that most metabolic studies have exclusively focused on metabolic disturbances and obesity, this study is significant because it points out the substantial effect of low muscle mass on mortality in addition to metabolic disturbances. Additionally, our findings provide a valuable perspective regarding the complex interplay among low muscle mass, frailty, and mortality. Low muscle mass emerges as a key determinant that exerts a significant direct effect on both all‐cause and CVD mortality and that indirectly contributes to the mortality risk by influencing frailty, as evidenced by its association with an increased frailty index score. Frailty acts as a mediator in the relationship between low muscle mass and mortality, accounting for a relatively modest portion of the total effect on CVD mortality. These findings underscore the substantial and predominantly direct effect of low muscle mass on mortality outcomes.

Our study has uncovered a compelling finding that warrants further investigation. We have observed an inverse relationship between low muscle mass and the occurrence of metabolic disorders, which may be attributable to the close relationship between muscle mass and body weight. A recent study by Matthew et al. demonstrated that all‐cause mortality was greater among individuals with low muscle mass in each BMI category, but the prevalence of low muscle mass decreased substantially with increasing BMI.^43^ Despite this protective association, our study has highlighted that the presence of low muscle mass is linked to a significantly elevated risk of mortality, indicating that its direct impacts are markedly adverse. This finding is consistent with recent research, which has shown that low muscle mass is associated with an increased risk of CVD and mortality. For instance, in a study of elderly Japanese women, low muscle mass was negatively associated with metabolic syndrome, yet it was linked to a high CVD risk.^44^ In a Greek cohort study, low muscle mass was found to significantly increase the 10‐year incidence of CVD in a multi‐adjusted regression model.^45^

There are some limitations to this study. First, the EWGSOP2 suggests the measurement of muscular strength or physical performance to define sarcopenia. However, as NHANES does not provide data on hand grip, chair stand test, and gait speed, we could not evaluate other functional parameters. Second, we could not consider the change in body weight and WC and the incidence of cardio‐metabolic comorbidities during the follow‐up period. Third, while a mediation analysis could examine the plausibility of relevant causal assumptions to ensure the causal interpretation of direct and indirect effect estimates, longitudinal and prospective studies should still be conducted to verify the results obtained in this work.

Nevertheless, this study has strength in that it is the first study to evaluate the effect of low muscle mass on mortality risk according to metabolic health and obesity status and to identify the mediation effect of metabolic disorder between low muscle mass and mortality. In particular, our findings revealed that low muscle mass was a significant predictor of increased all‐cause and CVD mortality risk, regardless of an individual's obesity or metabolic health status. It is worth noting that although individuals with low muscle mass had a lower rate of metabolic disorders, they still faced a markedly higher risk of mortality. These results underscore the importance of assessing and addressing skeletal muscle wasting as an independent risk factor for mortality, regardless of an individual's metabolic or obesity status. As such, our study highlights the need for additional research and intervention efforts aimed at improving muscle mass and function in individuals at risk for adverse health outcomes.

## Conflict of interest

All the authors declare that they have no competing interests.

## Supporting information


**Table S1.** Summary of 46 variables in the Frailty Index and their respective scorings.
**Figure S1.** Flowchart for final selection.
**Figure S2.** Subgroup analysis of adjusted hazard ratios of mortality according to low muscle mass, central obesity, and metabolic abnormalities.
**Figure S3.** Association between low muscle mass and mortality according to sex and age groups.

## Data Availability

The data for this study are available from the corresponding author upon reasonable request.
