# Supplementary material for: Association of low muscle mass and obesity with increased all‐cause and cardiovascular disease mortality in US adults
Source: J Cachexia Sarcopenia Muscle. 2023 Dec 18;15(1):240–54. doi: 10.1002/jcsm.13397 (PMC10834318; doi:10.1002/jcsm.13397)
Supplement: Supplementary file 1 — Table S1. Summary of 46 variables in the Frailty Index and their respective scorings. Figure S1. Flowchart for final selection. Figure S2. Subgroup analysis of adjusted hazard ratios of mortality according to low muscle mass, central obesity, and metabolic abnormalities. Figure S3. Association between low muscle mass and mortality according to sex and age groups. [file JCSM-15-240-s001.docx]

**Table S1**. Summary of 46 variables in the Frailty Index and their respective scorings

| Variable | Scoring |
| --- | --- |
| **Cognition** |  |
| 1. Confusion or inability to remember things | Yes=1, No=0 |
| Dependence |  |
| 2. Difficulty managing money | Difficulty=1, No Difficulty=0 |
| 3. Difficulty stooping, crouching, kneeling | Difficulty=1, No Difficulty=0 |
| 4. Difficulty lifting or carrying | Difficulty=1, No Difficulty=0 |
| 5. Difficulty preparing meals | Difficulty=1, No Difficulty=0 |
| 6. Difficulty walking between rooms on same floor | Difficulty=1, No Difficulty=0 |
| 7. Difficulty standing up from armless chair | Difficulty=1, No Difficulty=0 |
| 8. Difficulty getting in and out of bed | Difficulty=1, No Difficulty=0 |
| 9. Difficulty using fork and knife | Difficulty=1, No Difficulty=0 |
| 10. Difficulty dressing yourself difficulty | Difficulty=1, No Difficulty=0 |
| 11. Difficulty grasping/holding small objects | Difficulty=1, No Difficulty=0 |
| 12. Difficulty attending social event | Difficulty=1, No Difficulty=0 |
| **Comorbidities** |  |
| 13. Arthritis | Yes=1, No=0 |
| 14. Thyroid problems | Yes=1, No=0 |
| 15. Cancer | Yes=1, No=0 |
| 16. Coronary heart disease | Yes=1, No=0 |
| 17. Angina/ angina pectoris | Yes=1, No=0 |
| 18. Heart attack | Yes=1, No=0 |
| 19. Stroke | Yes=1, No=0 |
| 20. High blood pressure | Yes=1, No=0 |
| 21. Diabetes mellitus | Yes=1, No=0 |
| 22. Weak/failing kidneys | Yes=1, No=0 |
| **Hospital Utilization and Access to Care** |  |
| 23. General health condition | Fair, poor=1, Excellent, Very good, good=0 |
| 24. Number of prescribed medications | None=0, 1-4=0.5, 5 and more=1 |
| 25. Overnight hospital stays | Yes=1, No=0 |
| 26. Health compared 1 year ago | Worse=1, About the same, Better=0 |
| 27. Times receive healthcare over past year | None=0, 1-5=0.5, More than 5=1 |
| **Laboratory Values** |  |
| 28. Pulse rate (bpm) | 60-99=0, Other=1 |
| 29. Systolic blood pressure (mmHg) | 90-140=0, Other=1 |
| 30. Pulse pressure (mmHg) | 30-60=0, Other=1 |
| 31. Glycohemoglobin(%) | 0-5.7=0, >5.7=1 |
| 32. Fasting Glucose, serum (mmol/L) | 3·9-6·1=0, Other=1 |
| 33. Hemoglobin (g/dL) | M:13.5-18=0, Other=1; F: 12-16=0, Other=1 |
| 34. Red cell distribution width (%) | 11.6-14.6=0, Other=1 |
| 35. Mean cell volume (fL) | 80-96=0, Other=1 |
| 36. Platelet count SI (cells/µL) | 150-450=0, Other=1 |
| 37. Sodium (mmol/L) | 136-142=0, Other=1 |
| 38. Bicarbonate (mmol/L) | ≤28=0, >28=1 |
| 39. Total calcium (mmol/L) | 2-2.5=0, Other=1 |
| 40. Uric acid (µmol/L) | M: 240-510=0, Other=1; F: 160-430=0, Other=1 |
| 41. Blood urea nitrogen (mg/dL) | 3-20=0, Other=1 |
| 42. Creatinine (µmol/L) | M: 60-110=0, Other=1; F: 45-90=0, Other=1 |
| 43. Alkaline phosphatase (U/L) | ≤115=0, Other=1 |
| 44. Lactate dehydrogenase (U/L) | ≤190=0, Other=1 |
| 45. Total cholesterol (mmol/L) | ≤6.47=0, Other=1 |
| 46. Triglyceride (mmol/L) | <1.67=0, Other=1 |

**Figure S1.** Flowchart for final selection


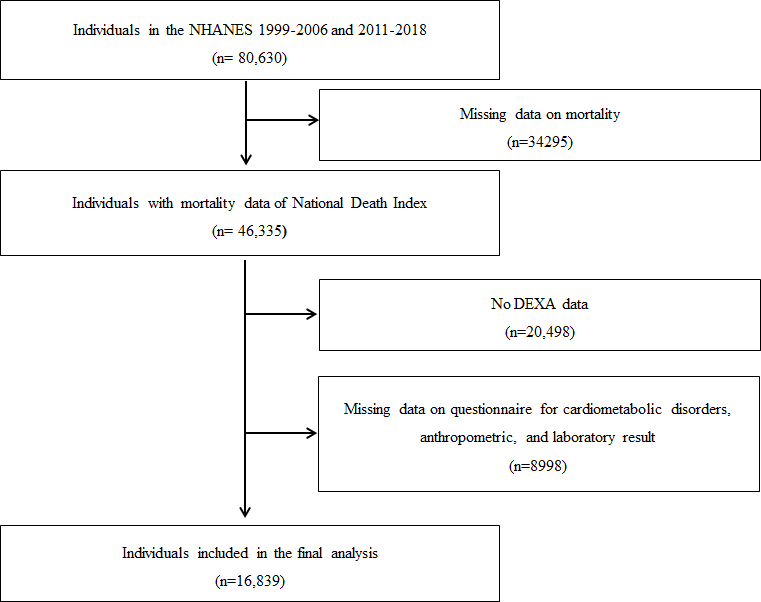


**Figure S2**. Subgroup analysis of adjusted hazard ratios of mortality according to low muscle mass, central obesity, and metabolic abnormalities

**
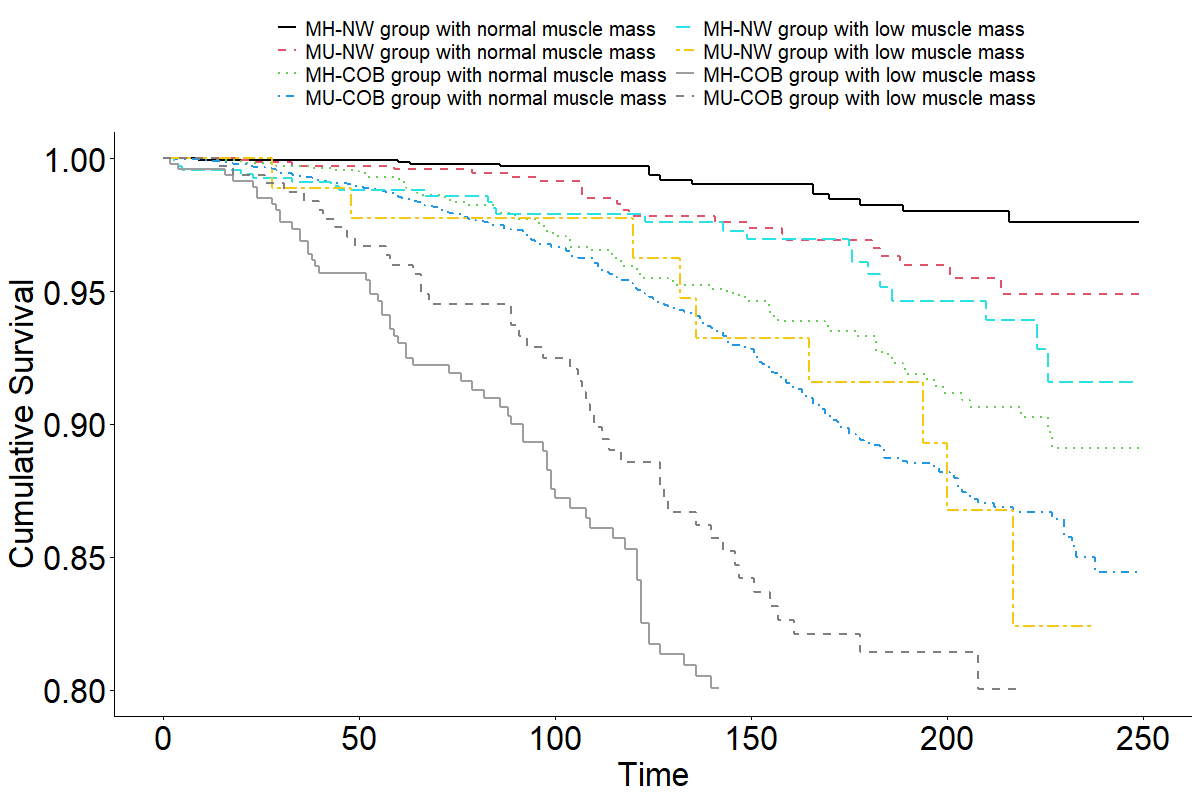
**(A) All-cause death

**
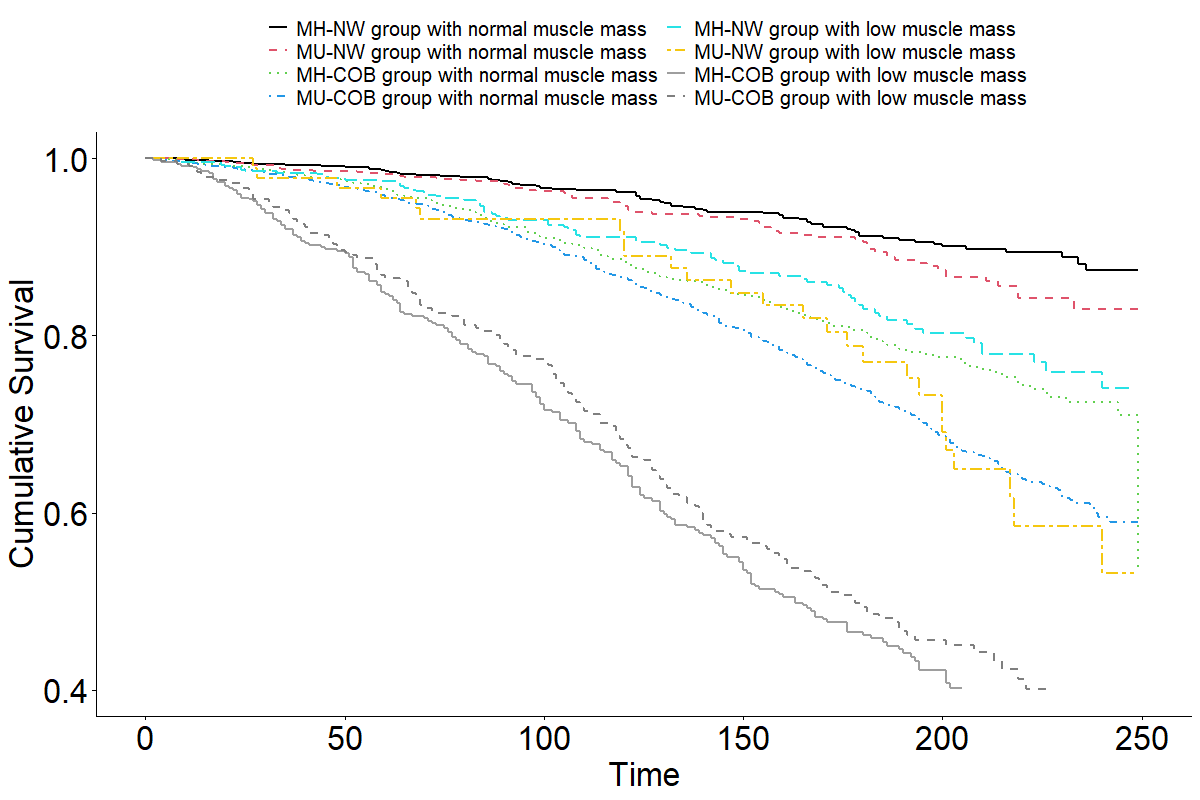
**(B) CVD death

**Abbreviation**: MH-NW, metabolically healthy with normal waist circumference; MH-COB, metabolically healthy with central obesity; MU-NW, metabolically unhealthy with normal waist circumference; MU-COB, metabolically unhealthy with central obesity

**Figure S3**. Association between low muscle mass and mortality according to sex and age groups.


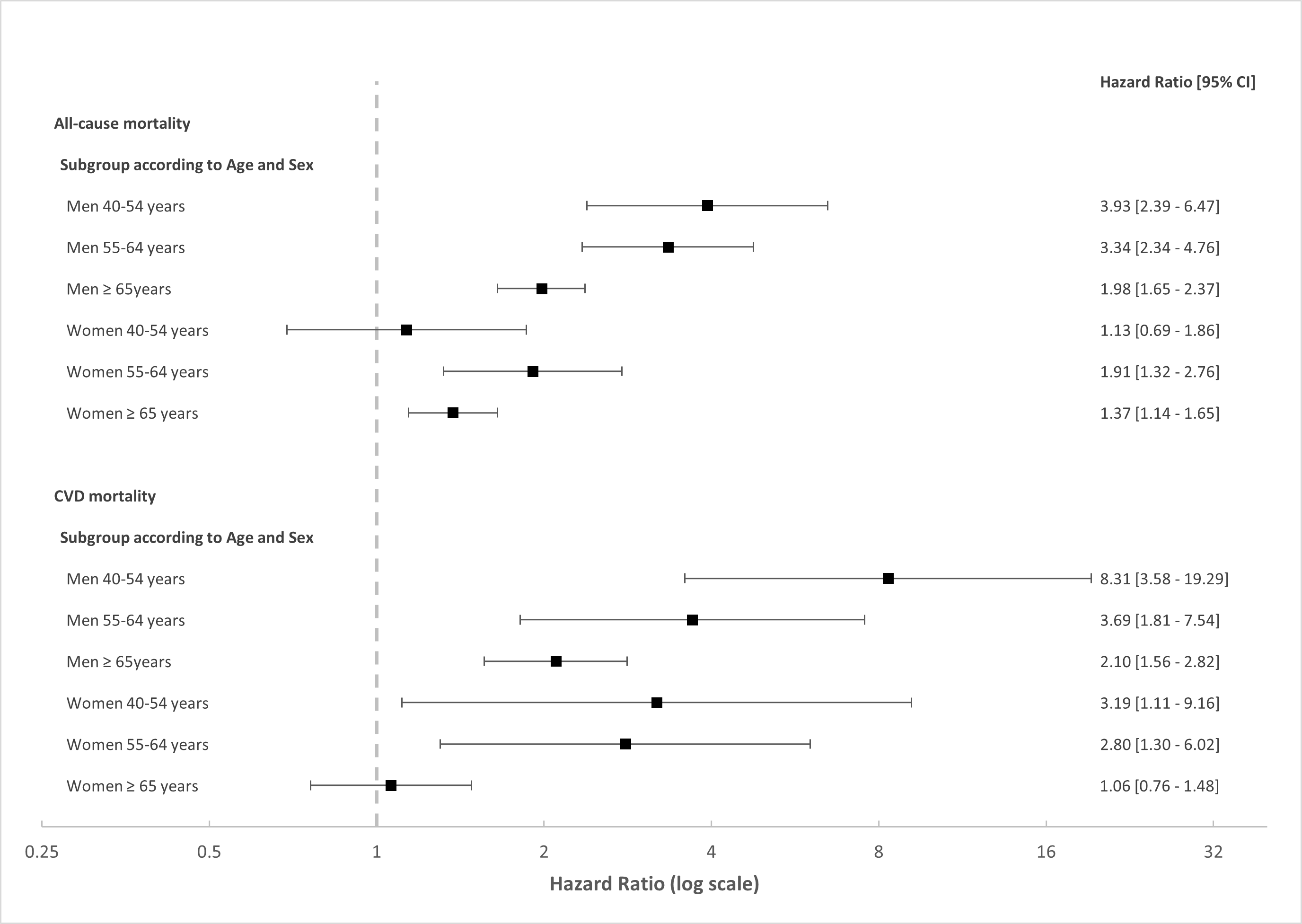


Adjusted for race, smoking status, alcohol consumption, eGFR, central obesity, history of cancer, HTN, DM, dyslipidemia, and past CVD events. **Abbreviations**: HR, hazard ratio; CI, confidence interval; COB, central obesity; DM, diabetes mellitus; CVD, cardiovascular disease.
